# Subarachnoid hemorrhage after transient global amnesia caused by cerebral venous congestion: case report

**DOI:** 10.1186/s12883-018-1042-3

**Published:** 2018-04-06

**Authors:** Yuta Maetani, Masahiro Nakamori, Tomoaki Watanabe, Hayato Matsushima, Eiji Imamura, Shinichi Wakabayashi

**Affiliations:** 1 Department of Neurology, Suiseikai Kajikawa Hospital, 1-1-23, Higashisendamachi, Naka-ku, Hiroshima, 730-0053 Hiroshima Japan; 2 Department of Neurosurgery, Suiseikai Kajikawa Hospital, 1-1-23, Higashisendamachi, Naka-ku, Hiroshima, 730-0053 Hiroshima Japan

**Keywords:** Transient global amnesia, Subarachnoid hemorrhage, Cerebral venous congestion, Valsalva maneuver, Straining

## Abstract

**Background:**

Transient global amnesia is reported to be caused by cerebral venous congestion. Internal jugular venous flow reversal in particular with the Valsalva maneuver leads to cerebral venous congestion. In addition, Valsalva maneuver can also induce subarachnoid hemorrhage. Transient global amnesia and subarachnoid hemorrhage might have common a pathophysiology in cerebral venous congestion.

**Case presentation:**

We report here the case of a 57-year-old woman who twice experienced convexal subarachnoid hemorrhage just after straining at stool following an episode of transient global amnesia. Digital subtraction angiography showed left temporal congestion. Left jugular vein ultrasonography revealed reflux with the Valsalva maneuver only in acute phase, indicating transient cerebral venous congestion.

**Conclusions:**

Subarachnoid hemorrhage followed by transient global amnesia indicates a common factor between them. Transient venous congestion is discussed in order to explain this rare phenomenon.

**Electronic supplementary material:**

The online version of this article (10.1186/s12883-018-1042-3) contains supplementary material, which is available to authorized users.

## Background

Transient global amnesia (TGA) is a clinical syndrome characterized by the sudden onset of reduced episodic memory. Several possibilities, such as cerebral ischemia and epileptic episode are suggested as the etiology of TGA, but the pathophysiology is still elusive. Internal jugular venous flow reversal that leads to cerebral venous congestion can contribute to TGA [1]. Particularly, the Valsalva maneuver leads to internal jugular venous flow reversal in patients with TGA [2].

Subarachnoid hemorrhage (SAH) is mainly caused by the rupture of a cerebral aneurysm or cerebral arteriovenous malformation, but sometimes the origin cannot be detected despite detailed examinations such as digital subtraction angiography (DSA). Valsalva maneuver that leads to cerebral venous congestion can also induce SAH [3].

Thus, TGA and SAH might be induced by venous congestion. However, there are few reports of the relation between TGA and SAH. Although TGA as a presentation of SAH has been reported, to the best of our knowledge, SAH after TGA has been never reported. We report the case of a patient with SAH after TGA following straining at stool.

## Case presentation

**Fig. 1:**
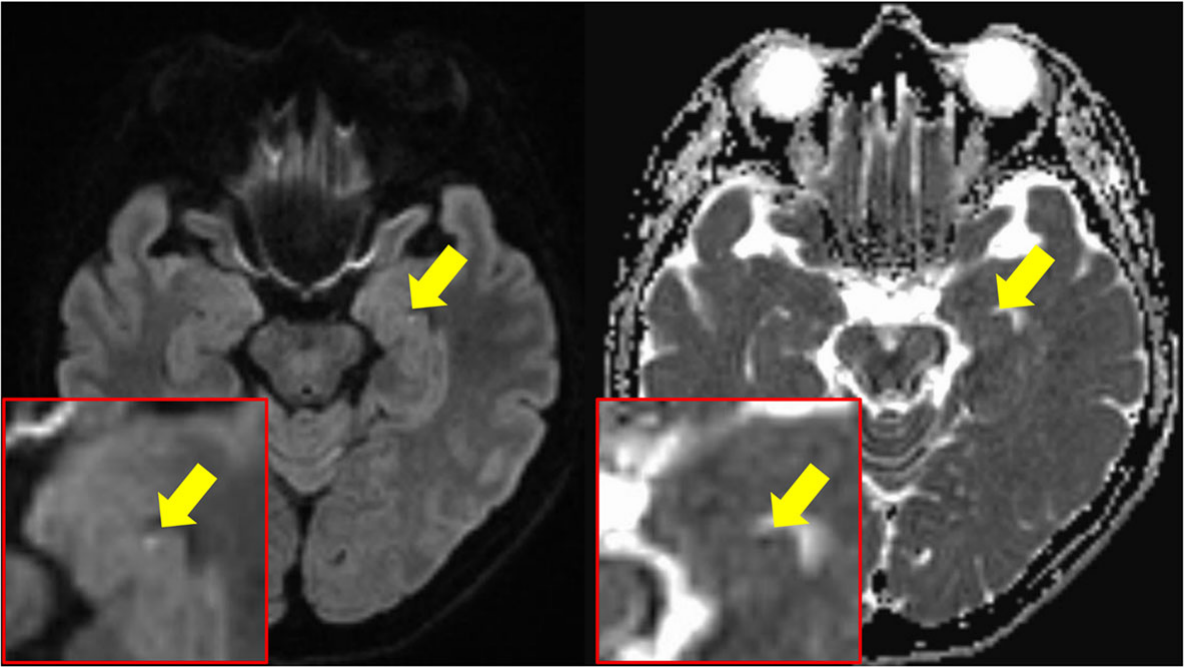
Brain magnetic resonance imaging (MRI) showed a high-density spot in the left hippocampus on diffusion-weighted imaging at the first admission

A 57-year-old woman was admitted to our hospital due to sudden-onset amnesia. She had no past medical history, except for dyslipidemia, had taken no medicine, including pills and nutritional supplements. She had neither smoked nor drank. She was an elementary school teacher. Before developing amnesia, she visited another elementary school for a workshop. She did not clearly remember the situation at the onset of TGA. At the first medical examination, she presented with no neurologic deficit, except for amnesia. Magnetic resonance imaging (MRI), electroencephalogram, and laboratory findings were normal. We diagnosed the patient with TGA. The patient’s symptom disappeared after about 6 hours. Diffusion-weighted imaging (DWI) carried out the next day revealed a high-density spot in the left hippocampus (Fig. 1), which indicated TGA. She was discharged two days after onset.

**Fig. 2:**
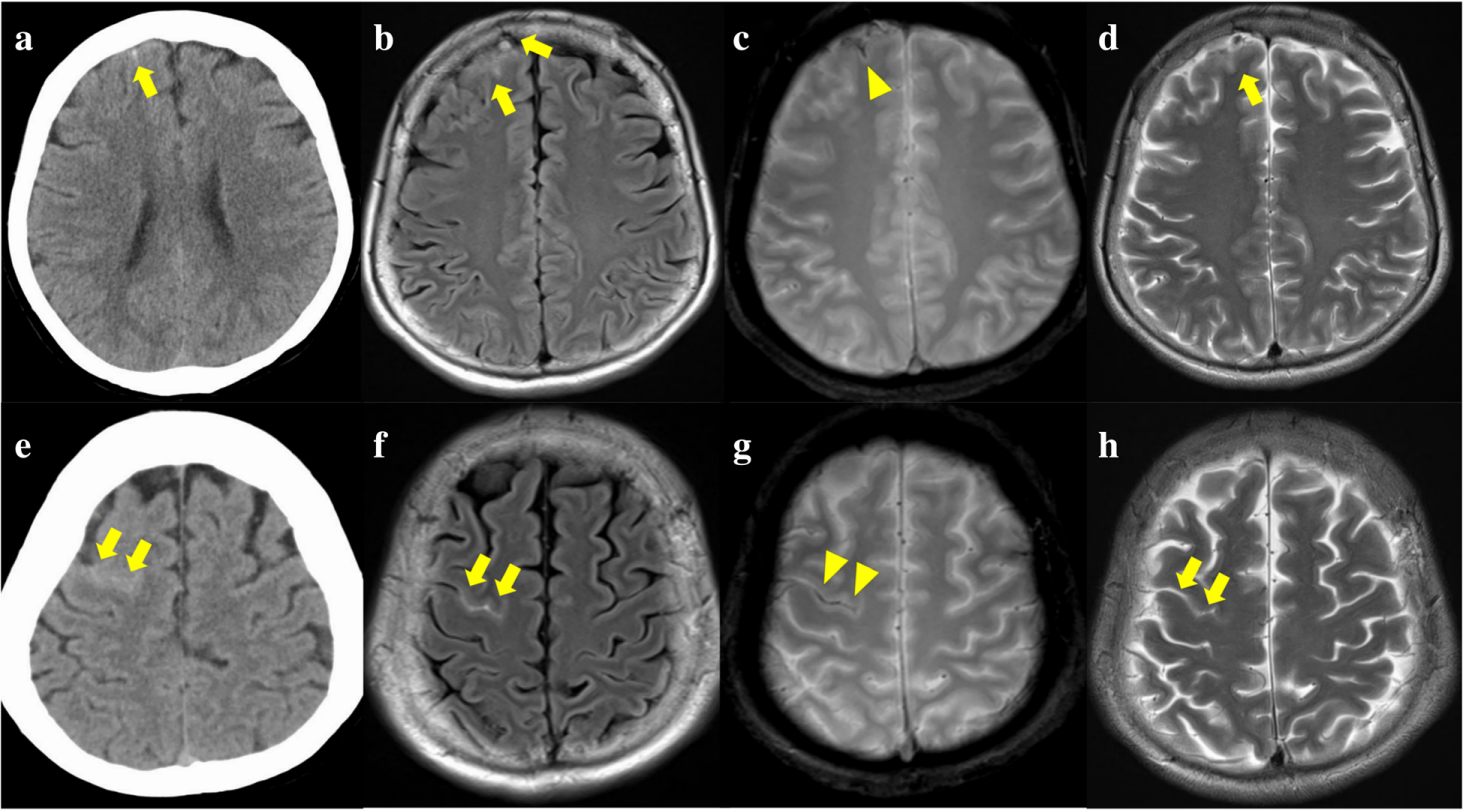
Head computed tomography and magnetic resonance imaging showed twice convexal subarachnoid hemorrhage on day1 of second admission (**a**-**d**), on day2 of second admission (**e-h**). Lesions have high density on computed tomography (**a**, **e**), a hyperintense signal on fluid-attenuated inversion recovery (**b**, **e**) and T2-weighted image (**d**, **h**) (arrow), and a hypointense signal on T2* susceptibility-weighted image (arrow head), which indicate not thrombosis but hemorrhage

**Fig. 3:**
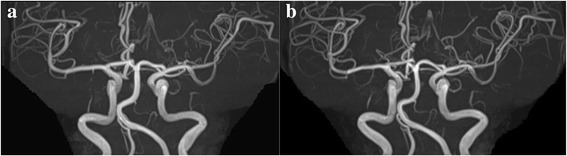
Magnetic resonance angiographies (MRA) showed no signs of reversible cerebral vasoconstriction syndrome. Images are presented from (**a**) the second admission, when the patient developed convexal subarachnoid hemorrhage, (**b**) discharge from the second admission. There was no change in MRA from the first admission, when the patient developed transient global amnesia, to four months after discharge from the hospital

**Fig. 4:**
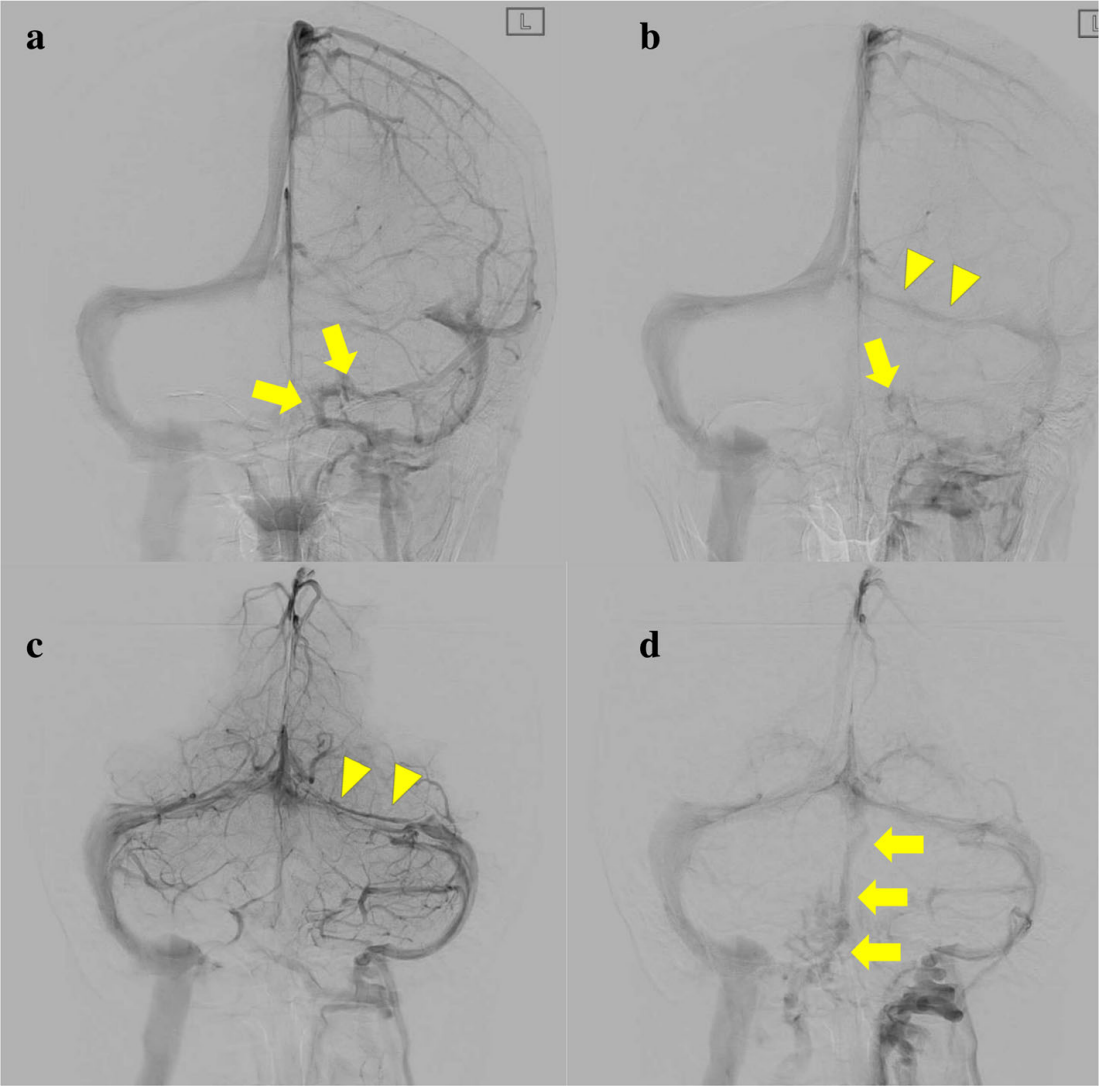
Digital subtraction angiographies are shown. **a** Left carotid artery angiography in the late venous phase, (**b**) in the last part of the venous phase. **c** Left vertebral artery angiography in the early venous phase, (**d**) in the late venous phase. Cerebral venous blood flowed into inferior petrosal vein through sigmoid sinus (**a**), and subsequently flowed backward from the inferior petrosal vein into the cavernous (arrow) (**b**). Digital subtraction angiography revealed left hypoplastic transvers sinus (arrow head). Blood flow through left transvers sinus was delayed and not much due to left hypoplastic sinus (**b**, **c**). Venous blood flowed normally into bilateral transverse sinus in the early venous phase (**c**), and furthermore, into the basilar venous plexus due to left hypoplastic transverse sinus in the late venous phase (**d**)

Several hours after discharge, the patient had a sudden terrible headache. The patient had strained at stool just before onset. She came back to our hospital immediately. At the time of the second admission, the patient was alert and had a blood pressure of 140/88mmHg. Her heart rate and oxygen saturation were normal. She had a throbbing headache, but exhibited no signs of neurologic deficit, including amnesia. Computed tomography (CT) and MRI revealed convexal subarachnoid hemorrhage (cSAH) in the right frontal lobe (Fig. 2a). The signal in this lesion was hyperintense on fluid-attenuated inversion recovery and T2-weighted images, and hypointense on T2* susceptibility-weighted images (Fig. 2b-d). This indicates hemorrhage and not thrombosis. There were no signs of leukoaraiosis or microbleeds on MRI and no vessel abnormalities, such as aneurysms or arteriovenous fistula/malformation on magnetic resonance angiography (MRA) (Fig. 3) and magnetic resonance venography (MRV). Laboratory findings, including those regarding the coagulating system, were normal. On day 2 of the second admission, a strong headache re-occurred when the patient strained at stool. CT and MRI revealed another cSAH in the right frontal lobe (Figs. 2e-h). There were no findings such as aneurysms, anomalies, or sinus thrombosis on DSA. However, DSA revealed hypoplastic left transvers sinus and congestion in the left temporal lobe. Left carotid artery angiography in the last part of the venous phase revealed reflux from the inferior petrosal vein into the cavernous (Fig. 4a, b). Left vertebral artery angiography revealed that hypoplastic left sinus caused venous blood flow into supplementary path, that is, the basilar venous plexus in addition to normal flow into bilateral transverse sinus (Fig. 4c, d), which indicated the left temporal venous congestion. However, perfusion-weighted imaging did not show distinct congestion in the lesion of cSAH.

**Fig. 5:**
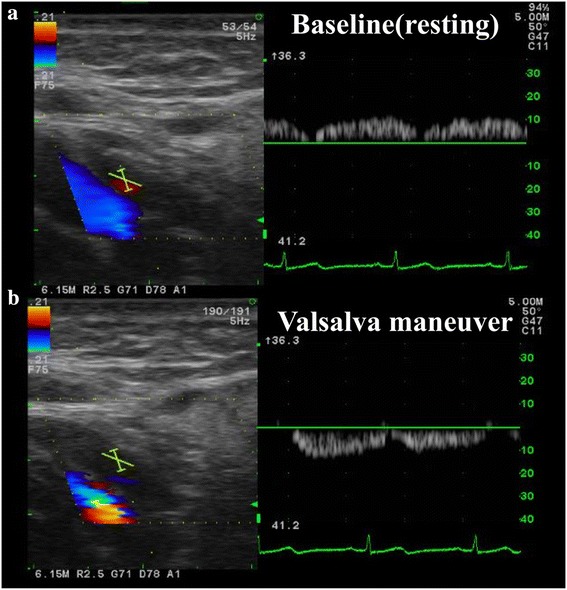
Left jugular vein echo results are shown. **a** Baseline (resting), (**b**) with Valsalva maneuver. Internal jugular reflux is observed only with Valsalva maneuver in the acute phase

Jugular vein ultrasonography revealed reflux only with the Valsalva maneuver in left internal jugular vein (Fig. 5). Reflux was confirmed according to a previous report [4]. The internal jugular venous valve was not found. The other examinations, such as electrocardiography, Holter electrocardiography, transthoracic echocardiography, carotid artery ultrasonography, leg venous ultrasonography, whole-body CT scan, and electroencephalogram were normal.

The headache steadily improved and the patient was discharged. Repeated brain imaging tests, including CT, MRI, MRA, and MRV indicated no relapse of cSAH, new lesion, reversible lesion, contraction, or thrombosis. The left internal jugular venous reflux following the Valsalva maneuver on ultrasonography disappeared four months later. Medical history and the test results are summarized in the Additional file 1.

## Discussion and Conclusion

Secondary TGA is likely to be caused by stroke, epilepsy, migraine, or abnormal cerebral venous reflux. CA-1 subfield of the hippocampus is related to TGA, and is affected by hypoxemia-induced metabolic stress, β-amyloid-induced neurotoxicity, and ischemia [1]. However, the predominant hypothesis is that TGA most commonly occurs due to abnormal cerebral venous drainage from the temporal lobes [1, 5]. Increased intrathoracic pressure, such as that caused by Valsalva maneuver, could trigger retrograde venous flow in patients with valve incompetence in the internal jugular veins. The resulting venous congestion would preferentially affect the function of the medial temporal lobes, thus triggering the anterograde amnesia [1]. In this case, DSA showed congestion in the temporal lobe.

Internal jugular venous flow reversal and internal jugular vein valve incompetency can contribute to TGA. Although not all patients with TGA show internal jugular venous flow reversal or internal jugular vein valve incompetency, this phenomenon is more common in patients with TGA than in controls or patients with transient ischemic attack [2]. Since the Valsalva maneuver causes internal jugular venous flow reversal in patients with TGA [2], in this case, it was not clear whether the patient performed the Valsalva maneuver before the onset of TGA. A high-density spot was found in her left hippocampus on DWI and left internal jugular venous flow reversal was noted with ultrasonography only during the acute phase. This was in agreement with a diagnosis of TGA.

However, a high-density spot on DWI also indicated ischemic stroke. TGA may be associated with right-to-left shunt, such as patent foramen ovale [6, 7]. We did not perform the right-to-left shunt study using transesophageal echocardiography or transcranial doppler. There were no abnormal findings such as thrombosis on leg venous ultrasonography and transthoracic echocardiography. Pulmonary arteriovenous fistula was ruled out by chest-body CT. We could not exclude paradoxical embolism, which is limitation, though results of our tests suggested that the patient was unlikely to have paradoxical embolism.

According to previous reports, TGA may not be associated with ischemic stroke. TGA patients have a much lower stroke risk than TIA patients [8]. On the other hand, the relation between TGA and SAH is not well documented [9]. Most studies reported amnesia as a presentation of SAH, i.e., secondary TGA is caused by SAH. To our knowledge, SAH after TGA has never been reported. Because TGA is a benign disease and recurrences are infrequent, the follow-up of the patients with TGA is stopped early. Thus, the relationship between TGA and SAH might be overlooked.

It is reported that cSAH may be caused by cerebral amyloid angiopathy (CAA), reversible cerebral vasoconstriction syndrome (RCVS), posterior reversible encephalopathy syndrome (PRES), or cerebral venous thrombosis (CVT) [10, 11]. In addition, patients ≤60 years frequently present a severe headache of abrupt onset with arterial narrowing on conventional angiograms, presumptively diagnosed with a primary vasoconstriction syndrome, while patients >60 years usually have temporary sensory or motor symptoms and are likely to present leukoaraiosis and/or hemispheric microbleeds and superficial siderosis on brain MRI, compatible with amyloid angiopathy [10]. In this case, CAA, RCVS, PRES, and CVT were not detected.

The Valsalva maneuver can induce SAH [3]. It is unlikely that the patient had headaches twice by chance just after straining at stool. Four months after admission, internal jugular venous flow reversal was not observed after the Valsalva maneuver and the patient did not have a headache, which suggested that transient venous congestion occurred at the onset of cSAH. Internal jugular venous flow reversal is seen in normal individuals, although it does not always lead to the development of TGA [1]. In this case, transient internal jugular venous flow reversal induced transient aggravation of venous congestion, which might have been pathological.

For SAH caused by CVT, the leading hypothesis is that secondary venous hypertension may trigger rupture of fragile superficial veins. The superficial veins have thin walls, which contain no smooth muscle fibers or no valves. Given obstructed drainage, these veins bridging the subarachnoid and subdural spaces can store blood and regulate cerebral venous stream [12]. This phenomenon may occur in TGA patients with internal jugular venous flow reversal due to venous congestion. Patients with TGA are reported to have hypoplastic venous sinus [13]. In this case, the left transverse sinus was hypoplastic. The frontal convexal veins might have been hypoplastic and fragile, which resulted in cSAH.

It is unclear why the patient had a hemorrhage twice in the right side. Left cerebral blood could not have avoided flowing into superior sagittal sinus due to left temporal congestion, which might have resulted in secondary right cerebral hemisphere congestion. Although we attempted to prove it, perfusion-weighted imaging did not reveal distinct increased blood flow in right convexal frontal lobe. Given congestion only on the surface of brain, it is difficult to prove the congestion. Further studies are required to test this hypothesis.

Few persons develop TGA or SAH, although venous congestion is common. Disease onset might be affected by venous structural deficits, such as vein valve incompetency and vessel wall vulnerability. Transient venous congestion may also contribute to disease onset.

The mechanism of the onset in this case was unique, as straining was the trigger. TGA may be attributed to abnormal venous flow. Therefore, TGA and cSAH might share the same precipitating factor, namely transient venous congestion.

## Additional file

Additional file 1: Timeline for CARE. (DOCX 49 kb)

## Abbreviations

CAA: Cerebral amyloid angiopathy
CSAH: Convexal subarachnoid hemorrhage
CT: Computed tomography
CVT: Cerebral venous thrombosis
DSA: Digital subtraction angiography
DWI: Diffusion-weighted imaging
MRA: Magnetic resonance angiography
MRI: Magnetic resonance imaging
MRV: Magnetic resonance venography
PRES: Posterior reversible encephalopathy syndrome
RCVS: Reversible cerebral vasoconstriction syndrome
SAH: Subarachnoid hemorrhage
TGA: Transient global amnesia
