# Supplementary material for: Subarachnoid hemorrhage after transient global amnesia caused by cerebral venous congestion: case report
Source: BMC Neurol. 2018 Apr 6;18:36. doi: 10.1186/s12883-018-1042-3 (PMC5889575; doi:10.1186/s12883-018-1042-3)
Supplement: Supplementary file 1 — Timeline for CARE. (DOCX 49 kb) [file 12883_2018_1042_MOESM1_ESM.docx]

| Dates | Relevant Past Medical History and Interventions | | |
| --- | --- | --- | --- |
|  | None | | |
| Dates | Summaries from Initial Follow-up Visits | Diagnostic Testing (including dates) | Interventions |
| Day 1-3 | Primary concerns and Physical Examination: only transient amnesia  Diagnosis: transient global amnesia (TGA) | MRI (Day2): high density spot in left hippocampus on DWI | spontaneous remission |
| Day3, 4 | twice convexal subarachnoid hemorrhages (cSAH) just after straining at stool following an episode of TGA | CT and MRI (Day3, 4): cSAH  DSA (Day6): Left hypoplastic transvers sinus and venous congestion in left temporal lobe  Ultrasonography (Day13): Left internal jugular venous reflux following the Valsalva maneuver | symptomatic treatment |
| Day 5- four months | no relapse of cSAH on repeated brain imaging  headache and reflux on ultrasonography following Valsalva maneuver disappeared (Four months) |  |  |
|  | Final outcome for this episode of care: TGA and cSAH might share the same precipitating factor; transient venous congestion. |  |  |

**Relevant Past Medical History: None**

**Four months**

**Follow up:** no headache, and no reflux on ultrasonography

**Follow up:** no relapse of cSAH on repeated brain imaging

**Resolution of the Episode of Care:**

TGA and cSAH might share the same precipitating factor, transient venous congestion.

**Day 1**

**Day 2**

**Day 3, 4**

**Second admission**

**First admission**

**Diagnosis:** cSAH induced by venous congestion

**Diagnostic Evaluations:**

convexal subarachnoid hemorrhage (cSAH) on CT

Left hypoplastic transvers sinus and venous congestion in left temporal lobe on DSA

Left internal jugular venous reflux following the Valsalva maneuver on ultrasonography

**Physical Examination:** only amnesia

**Physical Examination:** only headache

**Current Illness:** twice headache after straining at stool

**Diagnosis:** Transient global amnesia

**Diagnostic Evaluations:**high density spot in left hippocampus on DWI

**Current Illness:** transient amnesia
